# Should autologous HSCT be a first-line option in highly active MS? Commentary

**DOI:** 10.1177/13524585261439675

**Published:** 2026-04-26

**Authors:** Anna Karin Hedström

**Affiliations:** Department of clinical neuroscience, Karolinska Institutet, Stockholm, Sweden; Department of neurology, Karolinska University hospital, Stockholm, Sweden

Whether autologous hematopoietic stem cell transplantation (AHSCT) should be considered a first-line option in highly active multiple sclerosis (MS) depends less on its efficacy, which is increasingly difficult to dispute, and more on how its evidence compares with that of existing high-efficacy therapies. The accompanying articles illustrate how this question is framed differently depending on whether priority is given to biological plausibility or standards of evidence.

The argument in favor of early AHSCT is grounded in a clear biological rationale. Highly active MS is characterized by intense inflammatory activity that may drive irreversible central nervous system injury early in the disease course. From this perspective, AHSCT, through substantial immunoablation followed by immune reconstitution, offers a strong opportunity to alter the disease trajectory at a stage when therapeutic impact is likely to be greatest. Observational and comparative studies suggest that AHSCT can achieve durable suppression of disease activity, often exceeding that observed with high-efficacy disease-modifying therapies (DMTs).^1^

However, the counterargument highlights important limitations. The concept of “highly active MS” remains inconsistently defined,^2^ and AHSCT itself encompasses a range of protocols with differing intensity and safety profiles.^2^ More importantly, the current evidence base does not yet support routine first-line use.^3^ Direct comparisons with contemporary first-line high-efficacy DMT strategies in treatment-naïve patients are limited, and it remains uncertain whether the greater suppression of inflammatory disease activity observed with AHSCT translates into superior long-term disability outcomes.^4,5^ Safety considerations, including treatment-related toxicity, infertility, secondary autoimmunity, and a small but non-negligible risk of mortality, remain central to clinical decision-making.

A binary resolution of this debate may not fully capture the clinical complexity. At present, the available evidence does not justify its broad adoption as standard first-line therapy in highly active MS. At the same time, restricting AHSCT exclusively to later lines of treatment may overlook a subset of patients with rapidly evolving disease, in whom early, intensive intervention could plausibly confer long-term benefit. A pragmatic interpretation is to consider AHSCT as a potential first-line option in a narrowly defined subgroup of patients with clearly aggressive disease features and a high risk of early irreversible disability, provided that treatment is delivered within experienced centers and supported by careful patient selection and counseling. Such an approach should ideally be embedded within prospective data collection to further strengthen the evidence base.

Ultimately, the debate highlights the need for improved definitions of aggressive disease, standardization of transplant protocols, and comparative trials evaluating AHSCT against contemporary high-efficacy therapies in newly diagnosed patients. For now, the use of early AHSCT will continue to vary across settings, reflecting differences in the interpretation of evidence and available local expertise.

## Data Availability

Data sharing is not applicable to this article, as no data sets were generated or analyzed during the current study.
